# Amebicidal Effect of Adamantane–Azole Gold(I) Complexes: Cell Death Mechanisms and Synergistic Action with Chlorhexidine against *Acanthamoeba castellanii*

**DOI:** 10.1021/acsomega.5c11190

**Published:** 2026-01-28

**Authors:** Gabrieli Eduarda Israel, Gabriella da Rosa Monte Machado, Dayara Corrêa Matiola, Heveline Silva, Lisandra de Oliveira Silva, Maico Roberto Luckmann Rodrigues da Silva, Suellen dos Reis, Vitória Manoela Dambrós, Lílian Sibelle Campos Bernardes, Evelise Maria Nazari, Maria Cláudia Santos-Silva, Mário Lettieri Teixeira, Karin Silva Caumo

**Affiliations:** † Laboratório de Investigação Aplicada a Protozoários Emergentes (LADIPE), Programa de Pós-Graduação em Farmácia, Centro de Ciências da Saúde, 28117 Universidade Federal de Santa Catarina, Florianópolis, SC 88040-900, Brazil; ‡ Laboratório de Síntese e Interações Bioinorgânicas (SibLab), Departamento de Química, Instituto de Ciências Exatas, 28114 Universidade Federal de Minas Gerais, Belo Horizonte, MG 31270-901, Brazil; § Laboratório de Oncologia Experimental e Hemopatias (LOEH), Programa de Pós-Graduação em Farmácia, Centro de Ciências da Saúde, Universidade Federal de Santa Catarina, Florianópolis, SC 88040-900, Brazil; ∥ Departamento de Biologia Celular, Embriologia e Genética, Centro de Ciências Biológicas, Universidade Federal de Santa Catarina, Florianópolis, SC 88040-900, Brazil; ⊥ Laboratório de Química Farmacêutica Medicinal, Programa de Pós-Graduação em Farmácia, Centro de Ciências da Saúde, Universidade Federal de Santa Catarina, Florianópolis, SC 88040-900, Brazil; # Laboratório de Farmacologia, 215034 Instituto Federal Catarinense, Rodovia SC-283, Fragosos, Concórdia, SC 89700-000, Brazil

## Abstract

*Acanthamoeba* spp. are free-living protozoa associated with severe infections such as amebic keratitis and granulomatous amebic encephalitis. The absence of effective treatments highlights the need for new bioactive molecules targeting both trophozoite and cyst forms. This study evaluated the anti-*Acanthamoeba* activity of adamantane-azole gold­(I) complexes (C1 and C4) and their interaction with thioredoxin reductase (TrxR). Complexes C2, C3, and C4 exhibited potent amoebicidal activity with IC_50_ values of 0.12, 14, and 6.2 μM, respectively. They disrupted the cell cycle and induced phosphatidylserine exposure, while C4 also triggered mitochondrial depolarization. Ultrastructural alterations, synergy with chlorhexidine, and absence of toxicity in vitro and in vivo models were observed. Molecular docking confirmed TrxR as a potential target. These findings demonstrate the therapeutic promise of C2, C3, and C4 gold­(I) complexes against *Acanthamoeba* spp. infections, combining potent activity with minimal toxicity and supporting further investigation of their mechanisms of action.

## Graphical abstract

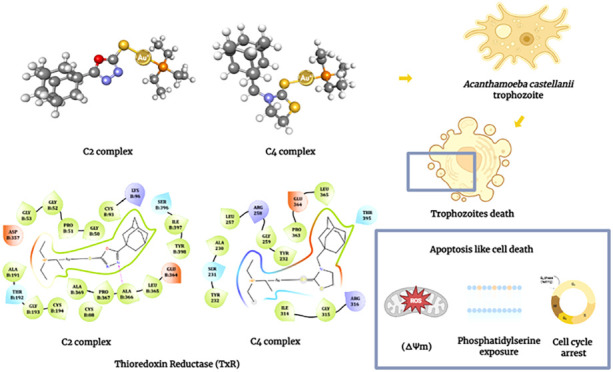

## Introduction

*Acanthamoeba* spp. are unicellular protozoa belonging to the free-living amoebae (FLA) group. Their role as etiological agents of infectious diseases such as granulomatous amebic encephalitis (GAE) and *Acanthamoeba* keratitis (AK) has increased the medical interest in recent years.^1,2^ GAE is a subacute, opportunistic infection of the central nervous system (CNS), which is fatal in more than 90% of cases,^3,4^ while AK is an ocular infection that mainly affects contact lens users.^5^ The latter is associated with poor hygiene practices and can lead to visual impairment and blindness if not diagnosed and treated promptly.^6,7^

*Acanthamoeba* spp. are well-adapted organisms with the ability to transition between two morphological forms: the trophozoite, the infectant form, and cyst, the resistance form.^8^ This characteristic makes these protozoa extremely resistant to the available current treatment or even any treatment so far tested against this protozoan.^9^ Despite the lack of a specific drug, the current protocols for AK treatment are based on 0.02% chlorhexidine associated or not to 0.01% polyhexamethylene biguanide (PHMB).^10^ The GAE treatment is based mainly on the association between five or more antimicrobial agents, of which the most used are rifampicin, amphotericin B, fluconazole, and, occasionally, the off-label use of miltefosine.^4^ Nevertheless, these drugs are poorly effective, and, on the AK treatment, it could be very aggressive, causing some adverse effects and complications to the patient.^11^

Several metallocomplexes have been reviewed over the last decades, with promising results against protozoa, such as *Toxoplasma gondii*, *Plasmodium falciparum*, *Trypanosoma cruzi*, and *Acanthamoeba* spp.^12–14^ Auranofin is a gold-based compound, and there is knowledge about its activity against the amoeboflagellate *Naegleria fowleri* and *Acanthamoeba* spp.^15–17^ Some previous studies have shown that complexes coordinated to tertiary phosphines have their lipophilicity increased, which, in turn, increases the permeability through the cell membrane and improves their activity in some cases, even surpassing that of auranofin.^18^ Garcia and co-workers previously reported the in vitro inhibition activity of rat liver thioredoxin reductase enzyme for this adamantane-azole gold­(I) complexes, with inhibition rates of 51.6, 57.3, and 60.2% for compounds C2, C3, and C4, respectively.^19^ The synthesized complexes were designed from known bioactive scaffolds: the phosphine ligands triphenylphosphine and triethylphosphine, recognized thioredoxin inhibitors; adamantane derivatives with established anticancer activity; and the heterocyclic ligands 1,3-thiazolidines and 1,3,4-oxadiazolines, which exhibit antimicrobial, antifungal, and antihelminthic properties.^19,20^

Other studies investigating thioredoxin reductase in *A. castellanii* have revealed the characteristics and roles of its components in oxidative stress response.^21^ The catalytic redox mechanism of thioredoxin reductase depends on NADPH and FAD as reducing agents and cofactors, respectively, and involves cysteine residues positioned at strategic sites to enable thioredoxin (Trx) reduction. Auranofin exhibits potent inhibition of thioredoxin reductase, thereby affecting trophozoite viability and suggesting its potential as a future therapeutic strategy for amebic infections.

In this study, we investigated the amoebicidal activity of gold­(I) complexes with adamantane-derived ligands containing heterocyclic 1,3-thiazolidines or 1,3,4-oxadiazolines and tertiary phosphines, both alone and in combination with chlorhexidine, as well as their *in vitro* and *in vivo* toxicity profiles. Furthermore, molecular docking studies were performed to provide useful information about the molecular mechanism of action of C2 and C4 complexes in the thioredoxin reductase enzyme.

## Results and Discussion

### In Vitro Activity of Gold­(I) Complexes against the Trophozoite Stage

The knowledge that auranofin, in addition to its antirheumatic activity, has antimicrobial, antiviral, antiparasitic, and antitumoral properties has sparked interest in the various potential uses of the gold metal complexes.^22^ In this study, among the four gold­(I) complexes (C1–C4) analyzed against *A. castellanii*, C2, C3, and C4 showed significant amebicidal activity. The effects were evidenced by morphological alterations in the trophozoites after treatment, which became rounded and smaller in size. The gold­(I) complexes C2, C3, and C4 were as effective as chlorhexidine (CLX), both at the same and at lower concentrations. The IC_50_ values for trophocidal activity were 87 ± 24, 0.12 ± 4.6, 14 ± 2.0, and 6.2 ± 5.0 μM for C1, C2, C3, and C4, respectively (Table 1). The IC_50_ values for the metal salts alone were 0.46 and 10 μM, while the ligands OXA and TIA exhibited IC_50_ values >40 μM.

**Table 1:** In Vitro Anti-*Acanthamoeba castellanii* Trophozoite Activity (IC_50_), Cytotoxicity (CC_50_) in SIRC Cells, and Selectivity Index (SI) of the Gold­(I) Complexes and Chlorhexidine

Gold(I) Complexes	MIAC^a^ (μM)	IC_50_ ^b^ (μM)	CC_50_ ^c^ (μM)	Selectivity Index^d^ (SI)
C1		87 ± 24		
C2	100	0.12 ± 4.6	11 ± 8.0	94
C3	100	14 ± 2.0	32 ± 2.3	2.4
C4	100	6.2 ± 5.0	10 ± 4.9	1.7
CLX	200	10 ± 1.1	53 ± 3.6	5.3

a Minimum inhibitory amoebicidal concentration (MIAC).

b Data are the mean ± SD of at least three different experiments performed in triplicate.

c Ratio between IC_50_ of trophozoites/CC_50_ of SIRC cells.

d All results are expressed in μM (parentheses), except for SI values.

Loufouma-Mbouaka and co-workers previously evaluated the activity of auranofin against *Acanthamoeba* strain Neff, an environmental nonpathogenic isolate, and a clinical isolate from a case of AK. IC_50_ values obtained were 2.97 and 3.48 μM, respectively.^17^ These findings are consistent with those of Feng and collaborators, who reported IC_50_ values of 5.79 ± 1.02 μM to *Acanthamoeba castellanii* (ATCC 30010).^23^ Like auranofin, the gold­(I) metal complexes evaluated in this study are tertiary phosphine gold­(I) complexes. However, they differ in the presence of adamantane-derived ligands and a heterocyclic structure such as 1,3-thiazolidines or 1,3,4-oxadiazolines.^19^

Comparable to the results obtained with auranofin, the gold­(I) complex C4 showed an IC_50_ value of 6.2 ± 5.0 μM against *A. castellanii* genotype T4. The gold­(I) complex C2, in turn, demonstrated higher potency against the same strain with an IC_50_ of 0.12 ± 4.6 μM (Table 1). Furthermore, our results proved to be superior to those of a gold­(I) complex synthesized and evaluated by Feng and collaborators, which demonstrated an IC_50_ value of 13.04 ± 1.53.^23^ Interestingly, the precursor gold phosphine alone, chloro­(triethylphosphine)­gold­(I), and chloro­(triphenylphosphine)­gold­(I) also exhibited inhibitory activity against *A. castellanii*.

Nonetheless, some studies have shown that the uptake of these compounds by cells is 5 to 25 times lower than when they are associated with a lipophilic ligand.^18^ Therefore, the addition of an adamantane-derived ligand was intended to increase the lipophilicity of the compounds, thereby enhancing their cellular uptake. The 1,3-thiazolidine and 1,3,4-oxadiazoline moieties were associated with adding the effects, since it has previously demonstrated antimicrobial, antifungal, and antihelmintic properties.^24–28^ Another study evaluated gold­(I) thiotetrazolates, a class of compounds similar to those used in this work, and identified these derivatives as potent inhibitors of TrxR in tumor cells. Interestingly, however, the thiotetrazolate ligand alone did not exhibit antiproliferative effects against these cells as in this study.^29^

### Transmission Electron Microscopy (TEM)

TEM analysis was performed on the trophozoites of *A. castellanii* treated with the C2 and C4 complexes (Figure 1), revealing several ultrastructural alterations. Control trophozoites displayed the typical features of *Acanthamoeba* spp., including a nucleus with a prominent nucleolus, a contractile vacuole, and well-preserved cytoplasmic organelles such as mitochondria and the Golgi complex.^30^ Both treatments induced a thickening of the cytoplasmic membrane, which was more pronounced in C4-treated trophozoites.

**Figure 1:**
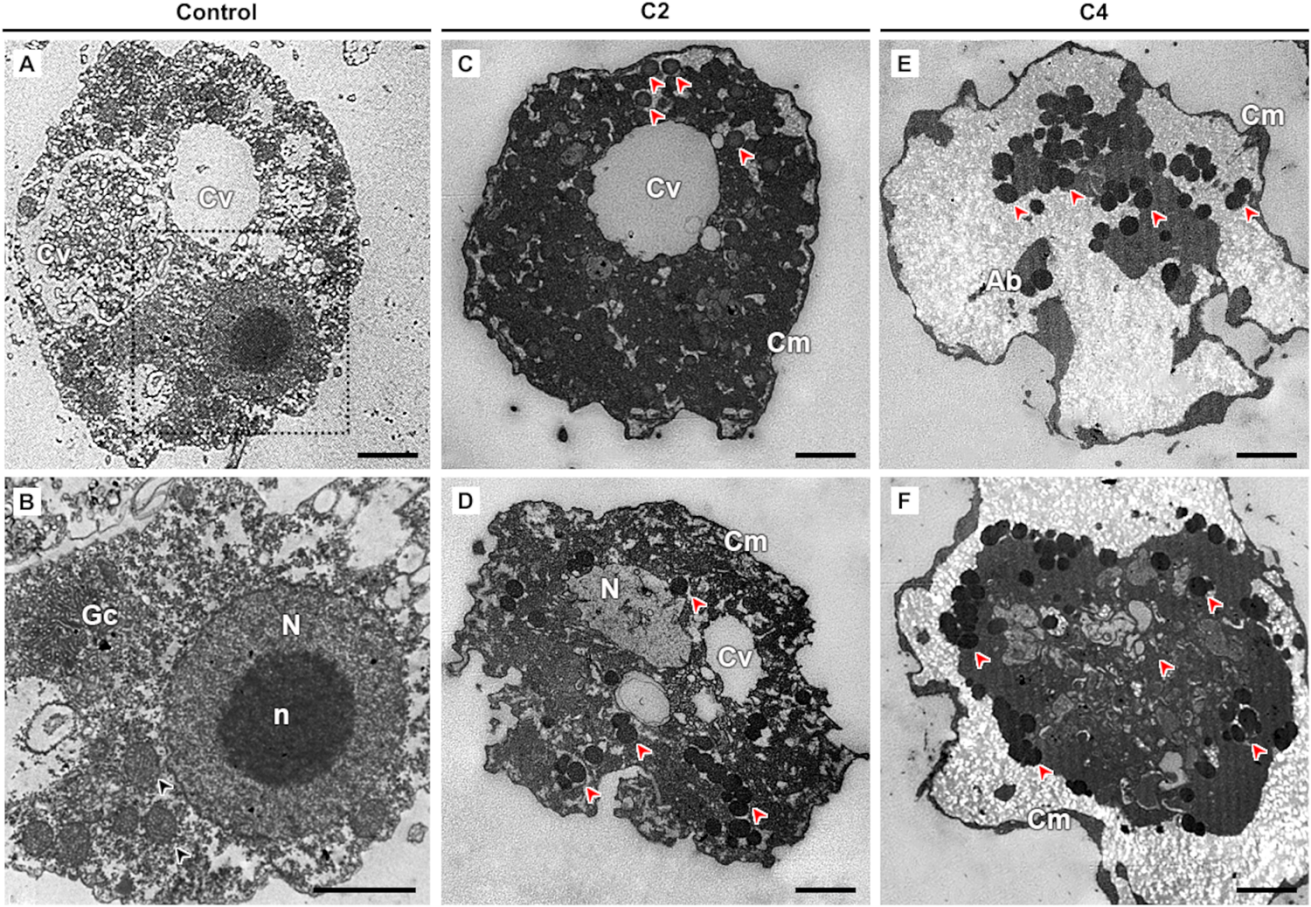
Ultrastructure of *Acanthamoeba castellanii* in transmission electron microscopy (TEM). (A, B), Negative control cultured in PYG medium (B), magnification of the dotted rectangle in (A). (C, D) Treatment with the C2 complex; (E, F) treatment with the C4 complex. Apoptotic body (Ab); cytoplasmic membrane (Cm); contractile vacuole (Cv); electron-dense granules (red arrowhead); Golgi complex (Gc); mitochondria (black arrowhead); nucleus (N); and nucleolus (n). Scale bars: 2 μm.

In C2-treated trophozoites, the cytoplasm was more electron-dense with abundant lipid droplets, while nuclear alterations included chromatin dispersion or clumping. The contractile vacuole remained evident, and mitochondria were still recognizable.

In contrast, C4-treated trophozoites exhibited an electron-translucent cytoplasm with no discernible organelles. Numerous lipid droplets were observed, which are predominantly associated with the nuclear membrane. The nucleus appeared condensed, apoptotic bodies were present, and the contractile vacuole was absent.

The ultrastructural alterations observed are indicative of pronounced cellular stress, leading to cell death. One hypothesis is that the electron-dense granules represent lipid droplets that specialize in lipid storage. Under the conditions of lipotoxicity, lipid droplets may exert a protective role by sequestering potentially harmful lipids and proteins. Nevertheless, the excessive accumulation of these unstable molecules, together with reactive oxygen species (ROS), can initiate lipid peroxidation, a process implicated in several modes of regulated cell death, including mitoptosis, ferroptosis, and apoptosis.^31^ An alternative explanation is that the granules correspond to lysosomes, which are crucial for maintaining cellular homeostasis through the degradation and recycling of macromolecules. Under stress or exposure to toxic compounds, however, lysosomal membrane destabilization may occur, resulting in the uncontrolled release of hydrolytic enzymes and proteases into the cytoplasm. This event can promote necrosis or apoptosis, aligning with the ultrastructural findings.^32^

### Gold­(I) Complexes Induce Cell Death in *A. castellanii*

The apoptosis assay was carried out using Annexin V-FITC, which enabled the evaluation of apoptotic cell rates by measuring the percentage of phosphatidylserine exposure, a phospholipid normally located on the cytosolic side of the plasma membrane. Phosphatidylserine externalization was observed at 12% ± 1.1, 5.9% ± 1.6, and 13% ± 2.4 for C2, C3, and C4 at 50 μM, respectively. Results are summarized in Figure 2.

**Figure 2:**
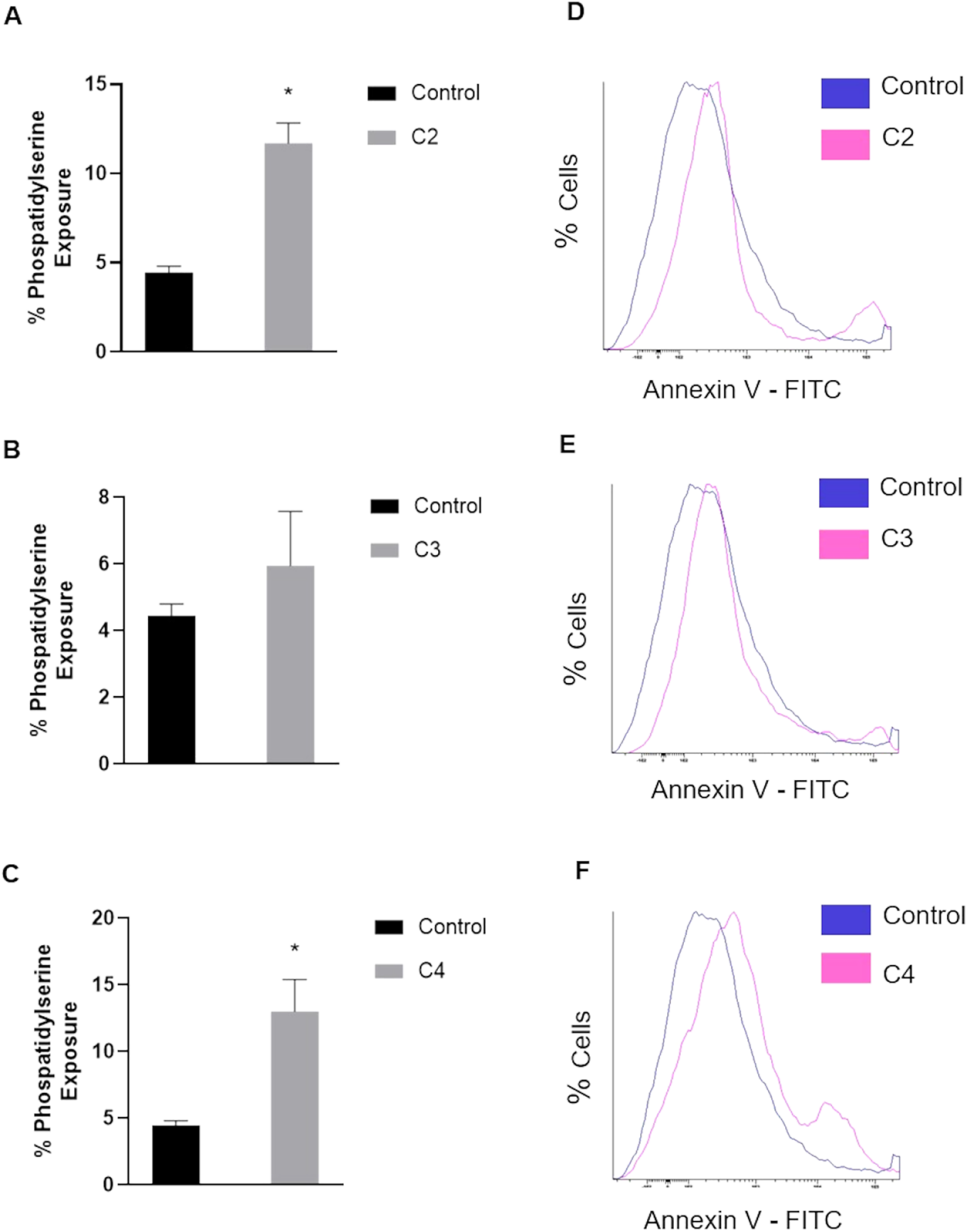
Annexin V-FITC assay to evaluate apoptosis in *A. castellanii*. The gold­(I) complexes C2, C3, and C4 were tested at 50 μM. (A–C) Percentage of phosphatidylserine exposure compared to the control. Data are presented as mean ± SD (*n* = 2). Statistical analysis was performed using an unpaired Student’s *t*-test. *p < 0.05 was considered significant when compared with control. (D–F) Histograms of cell density by Annexin V–FITC.

In cell cycle assays, in turn, at the tested concentration of 25 μM, the C2 and C4 complexes induced a discrete yet significant alteration in the cell cycle with an apparent reduction in the G2/M phase and a corresponding decrease in the G1 phase (Figure 3). The C3 complex was less effective at the same concentration, though it still exhibited a detectable difference compared to the control group. CLX, used as a positive control and considered a first-line treatment for AK, produced the most pronounced G1 arrest. Additionally, the synthesis (S) phase was significantly altered in both the positive control and the C2 complex at 25 μM.

**Figure 3:**
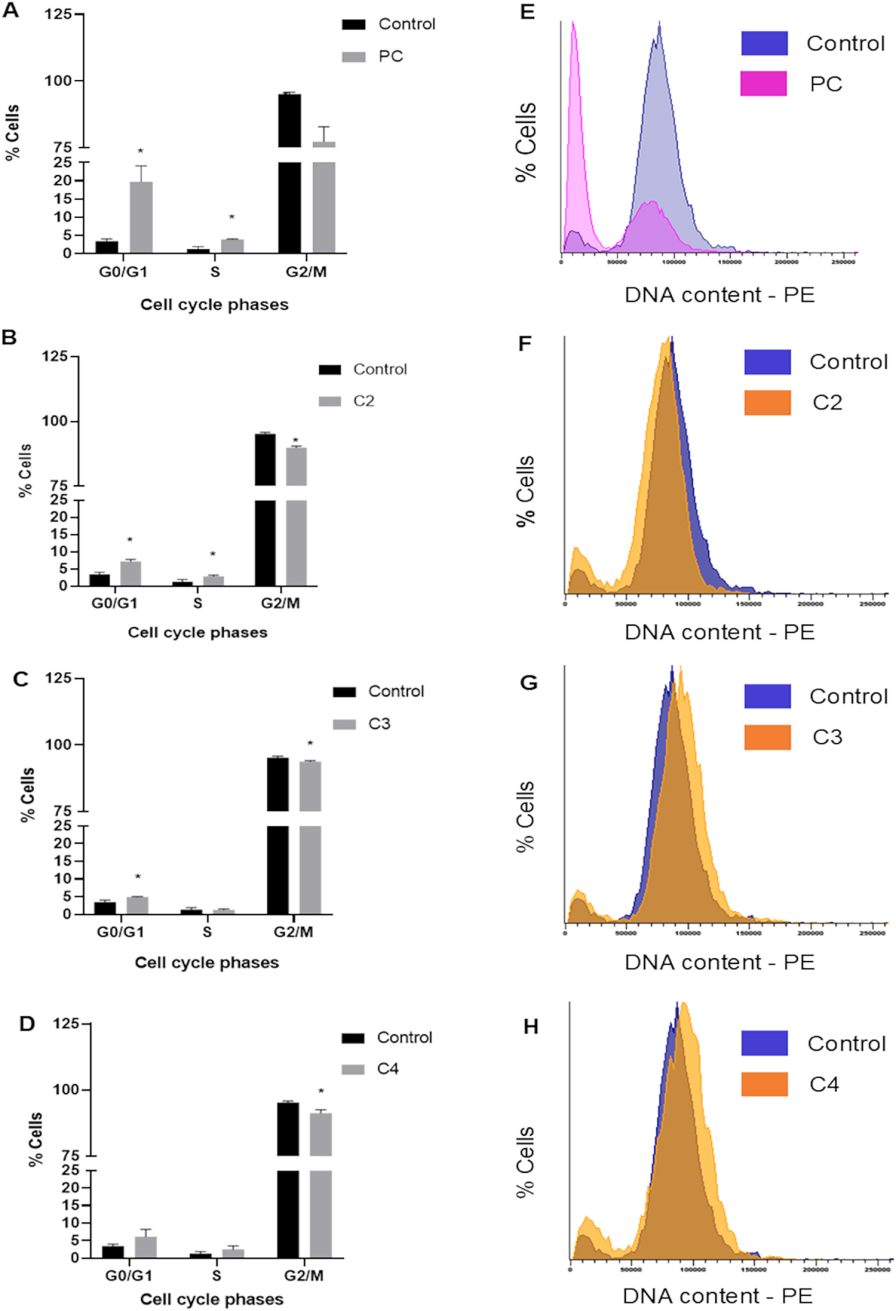
PI/RNase assay to evaluate the cell cycle of *A. castellanii*. Complexes C2, C3, and C4 were tested at a concentration of 25 μM. (A–D) Percentage of cells in each phase of the cell cycle (G1, S, and G2/M). PC: positive control. Data are presented as mean ± SD (*n* = 3). Statistical analysis was performed using an unpaired Student’s *t*-test. *p < 0.05 was considered significant when compared with control. (E–H) Distribution of cells according to DNA content, expressed as fluorescence intensity of PI-bound DNA.

The depolarization of the mitochondrial membrane potential (ΔΨ*m*), presented in Figure 4, was evaluated using a membrane potential-dependent fluorescent dye, where a reduction in fluorescence signal indicates a loss of ΔΨ*m*. Among the three gold­(I) complexes tested, only C4 at 50 μM interfered with *A. castellanii* mitochondria, leading to a significant decrease in ΔΨ*m*. Natamycin, previously reported to have anti-*Acanthamoeba* activity, was used as a positive control.^33,34^

**Figure 4:**
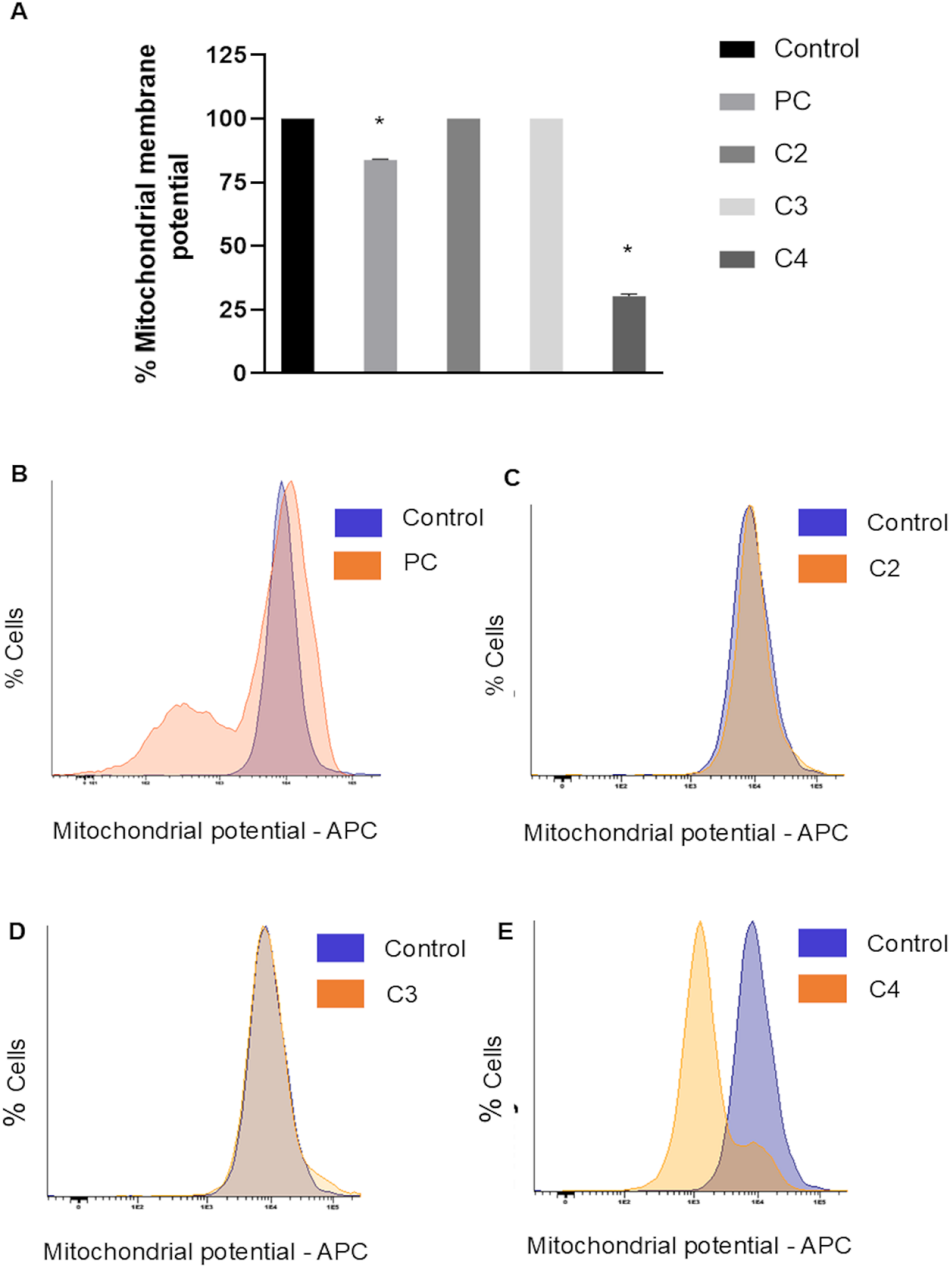
Mitoview 633-APC, mitochondrial membrane potential (ΔΨ*m*) assay to evaluate mitochondrial despolarization in *A. castellanii*. Complexes C2, C3, and C4 were tested at a concentration of 50 μM. (A) Percentage of mitochondrial membrane potential (ΔΨ*m*) in treated *A. castellanii*. PC: positive control. Data are presented as mean ± SD (*n* = 3). Statistical analysis was performed using an unpaired Student’s *t*-test. *p < 0.05 was considered significant when compared with control. (B–E) Histograms showing cell density distribution according to mitochondrial potential (APC fluorescence intensity).

The investigation of cell death mechanisms was performed using a combination of an apoptosis assay, cell cycle, and ΔΨ*m* assay. Inhibition of antioxidative enzymes in *Acanthamoeba* spp. leads to the accumulation of reactive oxygen species (ROS), resulting in cellular damage and triggering an apoptosis-like programmed cell death (PCD). This form of PCD differs from classical apoptosis observed in multicellular organisms, as its activation occurs through alternative pathways due to the absence of the canonical apoptotic machinery in protozoa.^35^ Nevertheless, apoptosis-like PCD in protozoa exhibits similar morphological and biochemical features, including chromatin condensation, nuclear DNA fragmentation, cell shrinkage, loss of mitochondrial membrane potential, formation of apoptotic bodies, and phosphatidylserine (PS) externalization.^36^ Despite mechanistic differences, both pathways ultimately lead to the same outcome: cellular death without necrosis and inflammation. Nonetheless, some cell death markers can appear in pathways other than apoptosis. For instance, phosphatidylserine (PS) exposure is observed in both apoptotic and necroptotic cell death. Although they share this feature, necroptosis is an inflammatory pathway triggered by mediators distinct from caspases, such as the kinase RIPK1 (activation of receptor-interacting protein kinase 1) and RIPK3 and the pseudokinase MLKL (mixed lineage kinase domain-like). Because these mechanisms may display overlapping markers, additional indicators of cell death were evaluated, as mitochondrial despolarization and cell cycle, in addition to transmission electron microscopy, to observe morphological alterations.^37,38^ Importantly, under these experimental conditions, no morphological evidence of extensive nonspecific necrosis was observed, supporting the conclusion that the responses detected were mechanistically informative rather than artifactual.

Apoptosis-like PCD has been extensively reported in *Acanthamoeba* spp., induced by various extracts and compounds.^39–42^ Among the three gold­(I) complexes tested, C4 was the most effective in triggering apoptosis-like PCD, as indicated by the investigated markers. The loss of ΔΨm observed in C4-treated cells may result from DNA damage caused by ROS accumulation and is likely associated with the release of pro-apoptotic proteins such as cytochrome c and endonuclease G. These proteins are potentially linked to caspase-like activity and nucleases, which are responsible for DNA degradation and the execution of apoptotic cell death.^35,43^ Other studies evaluated ROS-induced damage in *Acanthamoeba* spp. have also demonstrated that ROS leads to cell cycle arrest and mitochondrial membrane depolarization.^44,45^

The cell cycle analysis was conducted to evaluate the ability of the gold­(I) complexes to interfere with the cell cycle progression and cell division. In eukaryotic cells, the cell cycle is divided into four phases: G1 (the presynthetic gap), S (the synthesis phase, during which DNA is replicated), G2 (the postsynthetic gap, involving protein synthesis and cell growth), and M (the mitotic phase, where cell division occurs).^46^ In *Acanthamoeba* spp., these phases are similarly defined, but the duration spent in each phase differs from that of typical eukaryotic cells. According to previous studies, the cell cycle of *Acanthamoeba* spp. under asynchronous (exponential phase) and uninhibited growth conditions is characterized by approximately 90% of cells in the G2 phase, a lack of detectable G1 phase, and 8–10% of cells in the S/M phases.^47–49^ In this study, however, both gap phases (G1 and G2) were identified, consistent with the findings of Bínová and co-workers, who attributed this discrepancy to methodological differences among the aforementioned studies.^50^ Lee and collaborators reported cell cycle arrest in *Acanthamoeba* spp. following exposure to hydrogen peroxide (H_2_O_2_), an oxidative agent, observing a similar block at the G1/S transition.^45^ Similarly, Jha and collaborators demonstrated mitochondrial depolarization in *Acanthamoeba* spp. treated with juglone, which appeared to induce oxidative imbalance independent of ROS production.^44^ These findings support the hypothesis that adamantane-azole gold­(I) complexes inhibit TrxR enzymes, compromising the protozoan antioxidant defense and promoting ROS accumulation. The resulting oxidative stress is likely to act directly on DNA and mitochondria. Mitochondrial dysfunction, including reduced ATP synthesis, may contribute to cell cycle arrest.^36^

### Molecular Docking Studies with Gold­(I) Complexes

The molecular docking ligand target was first conducted on the FAD active site, and the best docking pose of C2 compound (Figure 5A) superposed to the cofactor, allowing hydrophobic interactions with Cys88 and Cys93 amino acids as π-alkyl and alkyl with the heterocyclic and adamantine rings. Moreover, the Leu365, Ala366, Ala369, Tyr398, and Pro510 residues have hydrophobic side chains, enabling the intermolecular interactions as alkyl and π-alkyl with the alkyl group bonded to the phosphorus atom and the adamantine ring, as presented in Figure 5B in 3D representation and in Figure 5C, in 2D. The heterocyclic ring of C2 has polar interaction as HBD and a hydrophobic interaction as π-alkyl with Arg366, sustaining the C2 stabilization in this binding site. On the other hand, the best docking pose for the C4 compound was not superposed to the cofactor (Figure 5D). In addition, the adopted pose enables the occupation of the access to the site of NADPH reductor agent by steric hindrance. This result provides new insights into potential enzyme inhibition. In this binding site, the amantadine ring established hydrophobic interactions as π-alkyl and alkyl with Tyr232, Leu365, and Pro363 residues (Figure 5D,E). Moreover, an alkyl hydrophobic interaction between Arg258 and the alkyl group bonded to the phosphorus atom from C4. Finally, the amino acids Arg316 and Tyr232 establish hydrophobic interaction as alkyl and polar as carbonic hydrogen with the heterocyclic ring, to the C4 stabilization in the cofactor active site. Results from the docking simulations indicate that the gold atom in the coordination complexes does not establish any intermolecular interaction with the active site amino acids.

**Figure 5:**
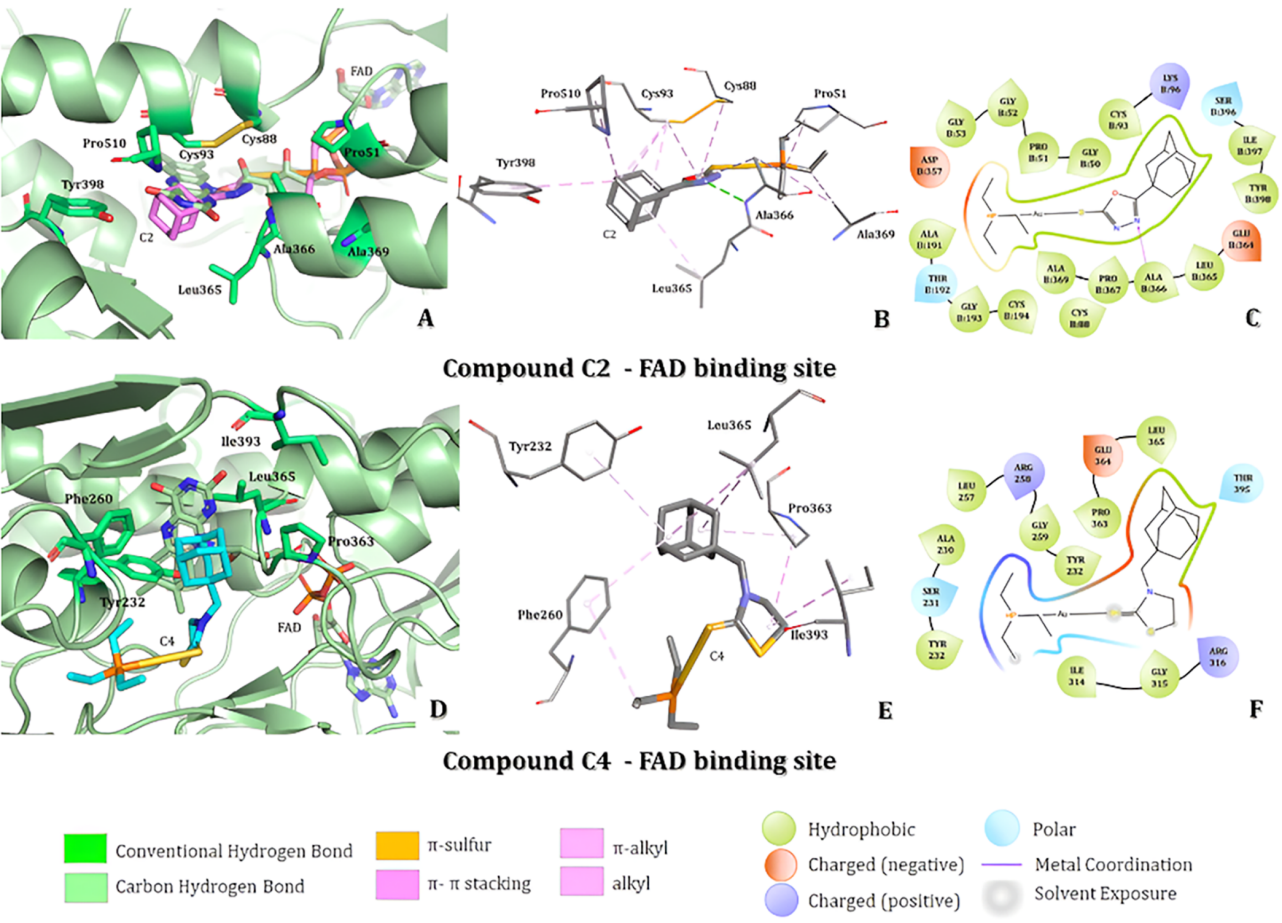
Molecular docking analysis in the FAD binding site of thioredoxin reductase from *Plasmodium falciparum* considering C2 and C4 gold­(I)-coordinate compounds. (A) Representation of C2 compound on the best docking pose and the binding site amino acids. (B, C) 3D and 2D representation of intermolecular interaction between C2 compound and the binding site amino acids. (D) Representation of C4 compound on the best docking pose and the binding site amino acids. (E, F) 3D and 2D representation of intermolecular interaction between C4 compound and the binding site amino acids.

The best docking pose to C2 in the substrate active site (Figure 6A) enables hydrophobic interaction with C-terminal Cys540 amino acid as alkyl with adamantine C2 portion, essential to enzyme TxR substrate reduction. With this docking pose, the compound C2 established hydrophobic interactions as π-π stacking and π-alkyl with the α-helix amino acid—as Phe143 and Arg139 with adamantine and heterocyclic ring. Moreover, the alkyl group bonded to the phosphorus atom interacted with Ile84 and Met146 amino acids as alkyl hydrophobic interactions, as presented in Figure 6B in 3D representation and in Figure 6C, in 2D. A similar outcome was observed with the docking pose for C4 compound (Figure 6D), enabling Cys540 amino acid interaction with the alkyl group bonded to the phosphorus atom as alkyl hydrophobic. Besides, the C4 binding site stabilization was achieved by the additional interactions with Phe143 as π-alkyl and π-sulfur with alkyl and heterocyclic ring of C4. Also, the alkyl interactions between Met146 and Ile84 residues and adamantine ring (Figure 6E, F).

**Figure 6:**
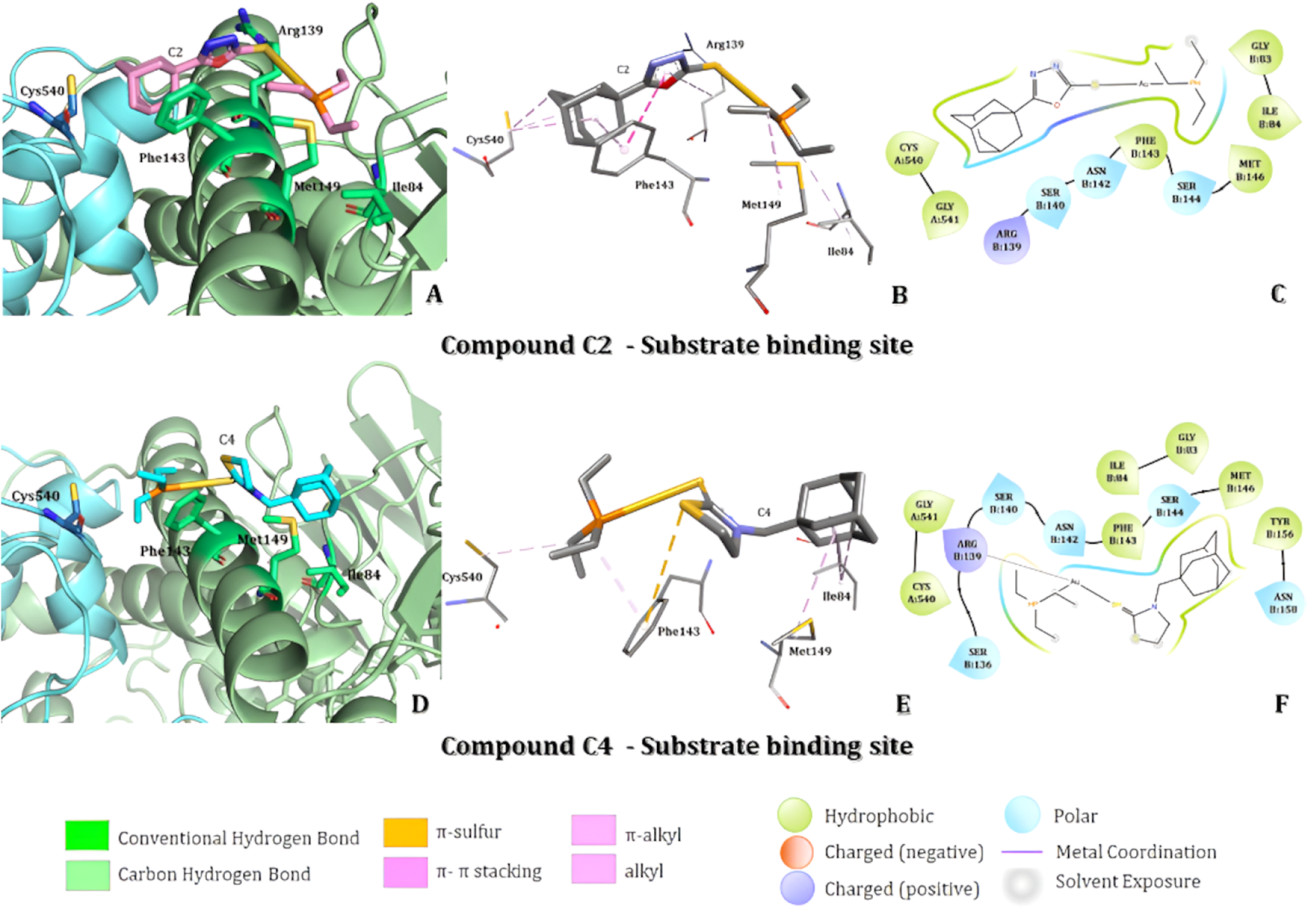
Molecular docking analysis in the substrate binding site of thioredoxin reductase from *P. falciparum* considering C2 and C4 gold­(I)-coordinate compounds. (A) Representation of the C2 compound on the best docking pose and the binding site amino acids. (B, C) 3D and 2D representation of intermolecular interaction between C2 compound and the binding site amino acids. (D) Representation of C4 compound on the best docking pose and the binding site amino acids. (E, F) 3D and 2D representation of intermolecular interaction between C4 compound and the binding site amino acids.

The molecular docking ligand target was conducted using the adamantane–azole gold­(I) compounds C2 and C4 (Table 2) due to the superior inhibitory activity against*A. castellanii* trophozoites and the synergism with chlorhexidine. The thioredoxin reductase (TrxR) enzyme from *P. falciparum* was investigated as a molecular target to facilitate comparisons with TrxR from the parasite *A. castellanii*. The C2 compound establishes interaction with Cys88 and Cys93 residues—at the FAD binding site—and with Cys540—at the substrate binding site—which are essential for the redox mechanism of action. The interactions with the cysteine residues are key factors in the catalysis of electronic exchange, through the formation and breaking of disulfide bridges, starting in the FAD cofactor region until the reduction of the substrate thioredoxin.^51^ This suggests a potential inhibitory effect of the C2 compound on thioredoxin reductase, mediated by hydrophobic interactions such as alkyl, π-alkyl, and π–π-stacking, and HBD polar interaction, which may contribute to the stabilization of the compound within the binding site. As a result, the C2 compound mimics the enzyme cofactor and substrate, thus preventing the formation of the disulfide bond, supporting the IC_50_ findings for this compound against *A. castellanii* trophozoites. The compound C4 adopted the docking pose in the NADPH access region, close to FAD, leading to a proposed inhibitor mechanism of action as steric hindrance. On the substrate binding site, the stabilization of C4 was achieved by alkyl, π-alkyl, and π-sulfur interaction with α-helix C-terminal amino acids, especially with Cys540. Therefore, based on the molecular modeling studies performed, the C2 and C4 compounds were theoretically predicted as potential inhibitors of thioredoxin reductase.^19^

**Table 2:** Evaluation of the Synergistic Effects of Chlorhexidine (CLX) in Combination with Gold­(I) Complexes (C2, C3, and C4)^a^

AmoebicidalConcentrations of Tested Compounds			
CLX	Gold(I) Complexes	FICI value	Description
MIAC/8	MIAC/8	0.375	synergistic
MIAC/4	MIAC/8	0.375	synergistic
MIAC/8	MIAC/4	0.65	indifferent
MIAC/4	MIAC/4	0.75	indifferent

a The MIAC values for CLX and the gold­(I) complexes were 200 μM and 100 μM, respectively. Fractional Inhibitory Concentration Index (FICI) interpretation*:* FICI ≤ 0.5 = synergism; 0.5 < FICI < 1.0 = indifference*;* FICI ≥ 1.0 = antagonism. The FICI values reported are identical for all three gold­(I) complexes (C2, C3, and C4).

### Checkerboard Assay

In this study, the synergistic potential between CLX and the gold­(I) complexes C2, C3, and C4 was evaluated. The minimum inhibitory amoebicidal concentrations (MIACs) for the three gold­(I) complexes were determined as 100 μM, while the CLX MIAC concentration was previously reported as 200 μM by da Silveira and collaborators.^14^ Using the checkerboard assay, combinations of CLX with each gold­(I) complex were tested, and the resulting reduction in trophozoites viability was assessed. Due to similar activity profiles of the three gold­(I) complexes, the FICI values were calculated and shared among them. The synergistic effect was considered when the FICI value is less than or equal to 0.5, and four synergistic concentrations were obtained for each complex with CLX (Table 2). The MIAC/8 and MIAC/4 of CLX correspond to concentrations of 25 and 50 μM, respectively, while MIAC/8 and MIAC/4 of complexes are 12.5 and 25 μM.

The synergistic effect of chlorhexidine combined with other compounds against *Acanthamoeba* spp. has been previously reported.^14,52^ Similar to findings of Mitsuwan and co-workers, our results demonstrated a synergistic interaction, maintaining high amoebicidal activity at concentrations 4- to 8-fold lower than the respective MIACs when used individually.^52^

### Evaluation of the Cytotoxicity of Gold­(I) Complexes on Rabbit Corneal Cells

The gold­(I) complexes exhibited dose-dependent cytotoxicity. Among the tested compounds, C3 demonstrated the lowest cytotoxicity, with cell viability rates of 96% at 10 μM and 78% at 50 μM. At the highest tested concentration (200 μM), C3 maintained a cell viability of approximately 56%. Compounds C2 and C4 preserved cell viability above 70% at 10 and 25 μM; however, at concentrations of 50, 100, and 200 μM, they showed marked cytotoxicity effects. Nonetheless, C2 showed a SI > 10, indicating a high selectivity for *A. castellanii* over the evaluated eukaryotic cell SIRC line (Statens Seruminstitut Rabbit Cornea). CC_50_ values are presented in Table 1. Garcia and co-workers also evaluated the cytotoxicity of these gold­(I) complexes against BHK-21 (Baby Hamster Kidney) cells, reporting CC_50_ values of 5.5 ± 0.1, 6.9 ± 0.8, and 5.8 ± 0.1 μM, for C2, C3, and C4, respectively, after 72 h of exposure.^19^

### Hen’s Egg Test on the Chorioallantoic Membrane (HET-CAM)

Beyond cytotoxicity assays in cell culture, the toxicity of the compounds was also evaluated using the HET-CAM assay, an in vitro method that assesses the potential irritancy of substances, primarily ocular irritancy.^53,54^ In this test, none of the evaluated gold metallocomplexes showed irritant potential on the chorioallantoic membrane of hen’s eggs at any of the time points analyzed. The negative control, 0.9% NaCl, demonstrated an irritation score (IS) of 0,20, while the gold­(I) complexes C1, C2, C3, and C4 showed IS values of 3.0, 3.1, 3.4, and 3.5, respectively. The positive control, 0,1 M NaOH, exhibited an IS of 19. IS values below 4.9 are classified as nonirritant, while 5.0 to 21 are classified as irritating.

### In Vivo Toxicity in *Tenebrio molitor* Larvae

An in vivo model using *T. molitor* larvae was employed to complement the toxicity profile of the gold­(I) complexes, which had previously shown no irritant effects at the highest concentrations tested. After 24 h of exposure, all gold metallocomplexes demonstrated a larval survival rate of approximately 70% at 200 μM. The mortality rate observed in larvae treated with the PBS control was similar to that of the treated groups, suggesting that mortality beyond 24 h may occur naturally rather than as a result of exposure to the gold compounds (Figure 7).

**Figure 7:**
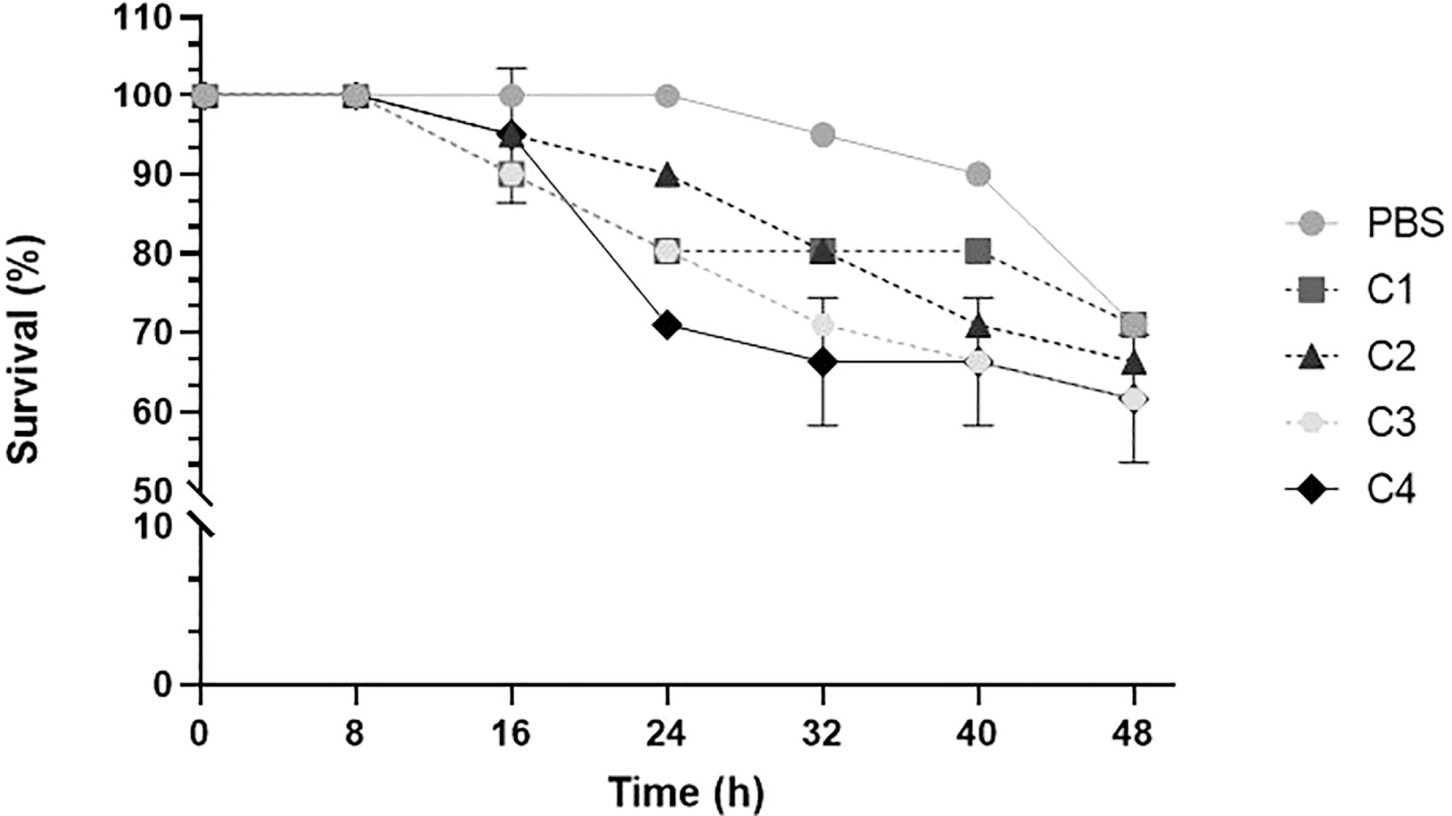
Kaplan–Meier plots for the survival curve of *T. molitor* mealworms treated with gold metallocomplexes C1–C4 at 200 μM each, and with phosphate buffer solution (PBS), in 48 h of exposition. The experiments were performed in triplicate. Data represent the mean ± SD *p < 0.5 when compared with the negative control.

The HET-CAM and *T. molitor* larvae models are particularly useful, as they are well suited for evaluating the toxicity of drug candidates with potential for treating ocular infections.^55,56^ These models also represent valuable alternatives to traditional animal testing, especially in the stages of screening molecules with pharmacological potential.

## Conclusion

The prospect of new drugs to treat *Acanthamoeba-*related diseases is increasingly urgent. The increase in the number of reported cases, associated with the death rates and reduction of quality of life, underscores the need for effective therapeutic alternatives. In this study, the gold­(I) complexes C2, C3, and C4 exhibited excellent activity against *A. castellanii*. These gold­(I) complexes achieved superior IC_50_ values when compared to other compounds and drugs, including auranofin (Ridaura), an FDA-approved gold-based drug. Additionally, molecular modeling studies revealed that compounds C2 and C4 established intermolecular interactions that contribute to the stabilization at both the cofactor and substrate binding sites of thioredoxin reductase, providing new insights into the enzyme’s mechanism of action. These findings support the activity of the compounds against *A. castellanii* trophozoites, with IC_50_ values below 10 μM. The cell death pathways were investigated, and some apoptosis-like markers were proven in *Acanthamoeba* post-treatment with these complexes, like loss of mitochondrial membrane potential (ΔΨ*m*) and arrest cell cycle. A synergistic effect between gold­(I) complexes (C2, C3, and C4) and the CLX was also observed, indicating their potential for future combination therapies. In conclusion, the adamantane–azole gold­(I) complexes demonstrated strong anti-*Acanthamoeba* activity, supporting their continued investigation and indicating that they may inform future research aimed at developing new therapeutic approaches for *Acanthamoeba* keratitis and granulomatous amebic encephalitis.

## Experimental Section

### *A. castellanii* and Cell Line Culture

For the amebicidal activity assay, the *A. castellanii* strain (ATCC 50492) genotype T4 was used in all experiments. Trophozoites were cultured in PYG medium [0.75% (w/v) protease peptone, 0.75% (w/v) yeast extract, and 1.5% (w/v) glucose] supplemented with penicillin–streptomycin (400 UI/mL each), and incubated at 30 °C for 120 h.^57^ For the cytotoxicity assay, Statens Seruminstitut Rabbit Cornea (SIRC) cells (ATCC CCL-60) were cultured in Minimum Essential Media (MEM) at 37 °C in a 5% CO_2_ atmosphere and maintained until 80% confluence.

### Synthesis of Ligands and Gold­(I) Complexes

The gold­(I) complexes with adamantine-derived ligands containing 1,3-thiazolidines or 1,3,4-oxadiazolines heterocycles, along with tertiary phosphine moieties, were provided by the Bioinorganic Synthesis and Interactions Laboratory (SIBLab/UFMG) (Table 3). The ligands and gold­(I) complexes were synthesized and characterized as previously reported by Garcia and co-workers.^19^

**Table 3:**
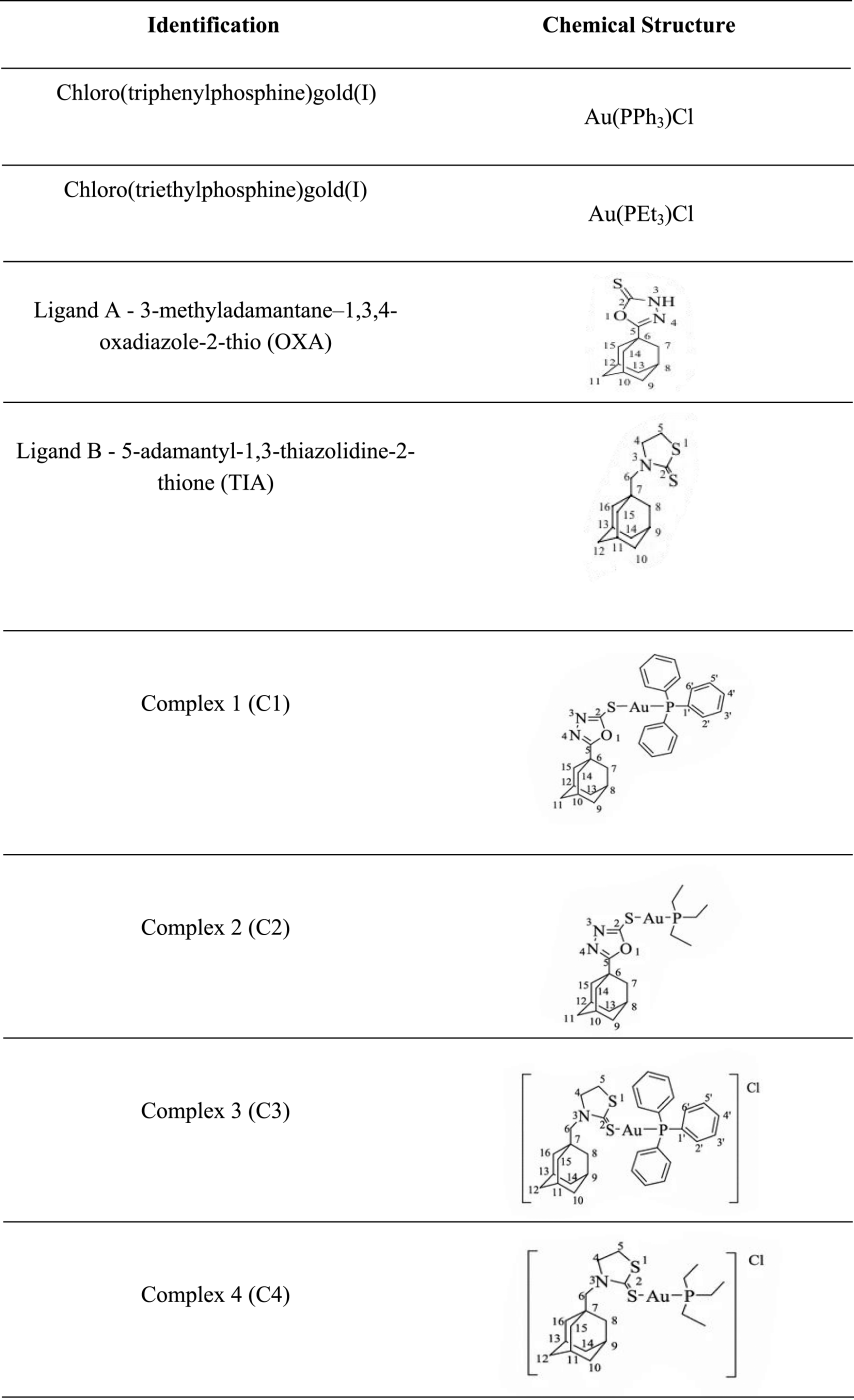
Molecular Structure of Adamantane–Azole Gold­(I) Complexes. Structures adapted from Garcia et al.^19^ ^a^

a Copyright 2016 Springer Nature.

### In Vitro Activity of Gold­(I) Complexes against Trophozoites

Trophozoites viability was evaluated using the Alamar Blue assay, following the protocols described by McBride and collaborators and Jha and collaborators.^44,58^ Adamantane–azole gold­(I) complexes C1, C2, C3, and C4 were tested at concentrations of 10, 25, 50, 100, and 200 μM for 24 h at 30 °C against *A. castellanii* trophozoites (8 × 10^5^ cells/mL), which were inoculated in a 96-well microplate. The gold salts, chloro­(triethylphosphine)­gold­(I) and chloro­(triphenylphosphine)­gold­(I), along with their respective ligands (OXA and TIA), were also tested at the same concentrations. Morphological changes in trophozoites were observed using an Olympus CKX53 optical microscope, and cell viability was determined using the Alamar Blue Cell Viability Reagent Assay (Thermo Fisher Scientific, Waltham, Massachusetts, EUA). Chlorhexidine (50 μM, Riohex 0.02%) and 0.1% DMSO were used as positive and negative controls, respectively. All experiments were performed triplicate and repeated independently three times. Results are presented as the percentage of trophozoites death. The minimum inhibitory amoebicidal concentration (MIAC) was defined as the concentration that inhibited >90% of the trophozoites. The 50% inhibitory concentration (IC_50_) was determined using GraphPad Prism 9.0 software by nonlinear regression analysis with a 95% confident interval.^52,59^

### Transmission Electron Microscopy (TEM)

Axenic cultures of *A. castellani* trophozoites (ATCC 50492) were maintained in PYG broth supplemented with penicillin-streptomycin (400 UI/mL each) and incubated at 30 °C. Upon reaching the logarithmic growth phase, the trophozoites were exposed to subinhibitory concentrations of MIAC/4, 25 μM, of gold­(I) complexes C2, and to C4 for 48 h. Therefore, the culture supernatant was removed, cells were centrifuged, and the resulting pellets were fixed in 2.5% glutaraldehyde and 4% paraformaldehyde in 0.1 M sodium cacodylate buffer (pH 7.4). After two washes with the same buffer, cells were postfixed in 1% osmium tetroxide (OsO_4_), under agitation for 1 h. Dehydration was carried out through a graded acetone series (50, 70, 80, 95, and 100%) for 20 min at each step. Finally, the cells were exposed to mixtures of acetone and Spurr resin (1:1, 1:2, and 2:1) for 12 h each. Subsequently, the samples were infiltrated with Spurr resin at room temperature for 24 h. Polymerization was then performed at 70 °C for 24 h. After polymerization, semithin sections (700 nm) were obtained using an ultramicrotome (Leica EM UC6, Germany) and stained with 1% toluidine blue. Ultrathin sections (60–70 nm) were then prepared, mounted on 3.05 mm Gilder copper grids (TAAB, U.K.), and stained with 5% uranyl acetate followed by 1% lead citrate. The samples were analyzed using a JEM-1011 transmission electron microscope (TEM) (JEOL, Japan) operating at 80 kV. This protocol was based on Ferreira and co-workers and Joo and co-workers, with modifications.^60,61^

### Apoptosis Assay

For the apoptosis assay, 1 × 10^6^ trophozoites/mL was inoculated in a 24-well microplate and exposed to the gold­(I) complexes C2, C3, and C4 at 50 μM for 24 h at 30 °C. This concentration corresponds to MIAC/2, based on MIAC of 100 μM for all complexes. After incubation, the well contents were collected, washed with phosphate-buffered saline (PBS), and centrifuged at 500*g* for 5 min. The pellet was resuspended in Annexin binding buffer (10 mM HEPES, 140 mM NaCl, and 2.5 mM CaCl2, pH 7.4) followed by the addition of annexin V-FITC. The mixture was incubated at room temperature (RT) for 15 min in the dark. The samples were analyzed using a BD FACSCanto II flow cytometer (Becton Dickinson Immunocytometry Systems) and analyzed with Infinicyt software version 2.0 (Cytognos). A total of 10,000 events were acquired per sample. Polyhexamethylene biguanide (PHMB) was used as positive control for apoptosis,^39^ and 0.1% DMSO was used as a negative control. The histograms are representative and illustrate a single experiment. The experiments were performed in independent duplicates, and the results are expressed as the mean and standard deviation (SD).

### Cell Cycle Assay

*A. castellanii* trophozoites (1 × 10^6^ cells/mL) were seeded into a 24-well microplate and exposed to the gold­(I) complexes C2, C3, and C4 at 25 μM for 24 h at 30 °C. This concentration corresponds to MIAC/4, based on an MIAC of 100 μM for all complexes. After incubation, the well contents were collected, washed with phosphate-buffered saline (PBS), and centrifuged at 500*g* for 5 min. After trophozoites immobilization, a cell cycle staining solution (1 M Tris–HCL, 70 U/mg RNase, 1 mg/mL propidium iodide, 100% Tween 80, and 5 M NaCl) was added, and the samples were incubated at room temperature for 10 min, protected from light. The samples were acquired using a BD FACSCanto flow cytometry II (Becton Dickinson Immunocytometry Systems) and analyzed using Infinicyt software version 2.0 (Cytognos). A total of 10,000 events were acquired per sample. The histograms illustrate a single experiment. The experiments were performed in independent triplicates, and results are expressed as the mean and standard deviation (SD).

### Mitochondrial Membrane Potential (ΔΨ*m*) Assay

*A. castellanii* strain at 1 × 10^6^ trophozoites/mL was added and exposed to the gold­(I) complexes C2, C3, and C4 at 50 μM for 24 h at 30 °C. This concentration corresponds to MIAC/2, based on MIAC of 100 μM for all complexes. Natamicyn was used as a positive control.^33,34^ Mitoview 633 (Biotium, Fremont, CA, USA), a mitochondrial membrane potential dye, was added according to the manufacturer’s instructions. The samples were incubated for 30 min and acquired using a BD FACSCanto II flow cytometry (Becton Dickinson Immunocytometry Systems) with Infinicyt software version 2.0 (Cytognos). A total of 10,000 events were acquired per sample. The histograms are representative and illustrate a single experiment. The experiments were performed in independent triplicates, and the results are expressed as the mean and standard deviation (SD).

### Docking Studies

Molecular docking studies were conducted using the GOLD v2024.3 package.^62^ The *P. falciparum* thioredoxin reductase crystal was selected as a molecular target in Protein Data Bank coded PDB 4J56, which has 2.37Å resolution. The applicability of the *P. falciparum* thioredoxin reductase crystal in molecular modeling studies is supported by unavailable crystal for *A. castellanii* enzyme, and by the identity of 47.51%, obtained by the alignment in BLASTp suite^63^ between *P. falciparum* TxR (PDB entry 4J56) compared to the sequence of *A. castellanii* TxR (Uniprot L8HG59).^64^ The structure has thioredoxin (substrate) and the FAD (cofactor) cocrystallized. Therefore, to explore the mode of interaction of the compounds as ligands, both the substrate and cofactor binding sites were taken into account.^65–67^ The docking grid was centered on the nitrogen atom located within the three-ring scaffold of FAD in monomer B, in the first study, and centered on the sulfur atom of the Cys540 residue in monomer A, in the second. The adamantane–azole gold­(I) corresponding to C2 and C4 compounds were drawn and optimized on BIOVIA Discovery Studio software v24.1, adding hydrogens and charges, and the geometry was optimized in Avogadro 12.0 software.^68^ The TrxR crystal structure was prepared using the Schrödinger Suite (Maestro Version 14.2, Release 2024) by adding hydrogen atoms according to standard protonation states at physiological pH, and by correcting missing side chains of amino acid residues.^69^ The grid was defined as a spherical region of 15 Å by redocking methodology, as the enzyme structure remained rigid during the simulation and the degrees of freedom of the ligand were preserved due to the generation of 100 docking poses. The score function with the best results was CHEMPLP according to the GOLD program genetic algorithm (GA). The intermolecular interactions obtained in the best docking poses were evaluated by Discovery Studio v24.1 software.^70^ This software, in combination with PyMOL, was also used for molecular visualization and image generation.

### Checkerboard Assay

Briefly, 8 × 10^5^ trophozoites/mL were inoculated into 96-well microplates. The synergistic effect of gold­(I) complexes (C2, C3, and C4) combined with chlorhexidine was evaluated at concentrations of 2× MIAC, MIAC, MIAC/2, MIAC/4, and MIAC/8 over 48 h at 30 °C. To CLX, the above concentrations correspond to 400, 200, 100, 50, and 25 μM, respectively, while complex concentrations, in the same order, are 200, 100, 50, 25, and 12.5 μM. The MIAC for each gold­(I) complex was determined from a preliminary dose–response screening. Cell viability was assessed using the Alamar Blue Cell Viability Assay (Thermo Fisher Scientific, Waltham, MA, USA) as previously described for da Silveira and collaborators.^14^ Chlorhexidine (Riohex 0.02%) served as a positive control, and 0,1% DMSO- treated trophozoites were used as a negative control. All experiments were performed in triplicate. The synergistic interaction was analyzed using the fractional inhibitory concentration index (FICI), calculated with eq 1, where FICI ≤ 0.5 indicates synergy, >0.5 and ≤4 indicate an indifferent effect, and >4 indicates antagonism.^71,72^

1 $$\mathrm{FICI}=\frac{\mathrm{MIAC}\;\mathrm{combination}\;\mathrm{of}\;\mathrm{compound}\;A+\mathrm{compound}\;B}{\mathrm{MIAC}\;\mathrm{compound}\;A\;\mathrm{alone}}+\frac{\mathrm{MIAC}\;\mathrm{combination}\;\mathrm{of}\;\mathrm{compound}\;B+\mathrm{compound}\;A}{\mathrm{MIAC}\;\mathrm{compound}\;B\;\mathrm{alone}}$$

### Cytotoxicity Evaluation on Corneal Cells

SIRC corneal cells were seeded at a density of 9 × 10^3^ cells/well in 96-well microplates and incubated at 37 °C in a 5% CO_2_ atmosphere until reaching 80% confluence. Cells were then treated with gold­(I) complexes (C2, C3, and C4) at concentrations of 10, 25, 50, 100, and 200 μM for 24 h. Cell viability was assessed using the MTT Cell Viability Assay (Thermo Fisher Scientific, Waltham, MA, USA) following the manufacturer’s protocol. Chlorhexidine (Riohex 0.02%) was used as a positive control, and 0.1% DMSO was used as a negative control. Results were expressed as the percentage of viable cells, including mean values and standard deviation (SD) from triplicate experiments. The 50% cytotoxic concentration (CC_50_) was calculated using nonlinear regression analysis with a 95% confidence interval. The selective index (SI) for *A. castellanii* was determined by the ratio of CC_50_ (SIRC cells) to IC_50_ (*A. castellanii*).

### Hen′s Egg Test on the Chorioallantoic Membrane (HET-CAM)

The irritant potential of the gold­(I) complexes was evaluated using the HET-CAM assay, following the guidelines established by Interagency Coordinating Committee on the Validation of Alternative Methods (ICCVAM).^54^ Fresh, white, fertile Lohmann eggs (Lohmann selected Leghorn, LSL) were incubated under controlled conditions at a temperature of 38–39 °C and relative humidity of 55–60% for 8 days. On the eighth day, the egg shell, around the airspace, was carefully removed with a rotary tool (Dremel, WI, USA) to expose the chorioallantoic membrane (CAM). A volume of 0.3 mL of each test solution was applied directly to CAM, with the following groups: negative control, 0.9% saline solution; positive control, 0.1 M sodium hydroxide (NaOH) and test group, gold­(I) complexes at 200 μM concentration. The CAM was observed for vascular reactions, including hemorrhage, lysis, and coagulation, at 30 s, 2 min, and 5 min postapplication. The irritation score (IS) was given according to the eq 2, from a scale from 0 to 4.9 as nonirritant (or practically no irritation) and from 5.0 to 21 as irritating (severe or extreme irritation).^73^

2 $$\mathrm{IS}=(\left(\frac{\left(301-\mathrm{hemorrhage}\;\mathrm{time}\right)}{300}\right)\times 5)+(\left(\frac{301-\mathrm{lysis}\;\mathrm{time}}{300}\right)\times 7)+(\left(\frac{301-\mathrm{coagulation}\;\mathrm{time}}{300}\right)\times 9)$$

### In Vivo Acute Toxicity Tests in Mealworms

*T. molitor* larvae (Coleoptera: Tenebrionidae) were anesthetized by cooling at 2 °C for 2 min. Then, 50 μL of each gold­(I) metallocomplex at 200 μM was applied in each larva, with support of a microsyringe into the hemocoel, at the second or third visible sternite above the legs, in the ventral portion. The larvae were incubated at 37 °C in Petri dishes containing a rearing diet. The number of dead larvae was recorded at 4 h intervals over 48 h. The assay was performed with groups of seven larvae in triplicate, resulting in 21 larvae for treatment. The assay followed de Souza and co-workers and Usui and co-workers.^74,75^

### Statistical Analysis

The experiments were performed in technical and biological triplicates. Data were recorded and analyzed using GraphPad Prism software (ver. 9.0.0; La Jolla, CA, USA). Results were expressed as the mean and standard deviation (SD). For multiple group comparisons (trophozoites viability and cytotoxicity), data were analyzed using one-way analysis of variance (ANOVA) followed by Turkey’s post hoc test. For comparisons between two groups (treatment vs control) in the assay of mitochondrial membrane potential, cell cycle distribution, and apoptosis, the Student’s *t*-test (unpaired, two-tailed) was applied. A significance level of *p* ≤ 0.05 was considered statistically significant when compared to the control group (untreated trophozoites).

## Supplementary Material



## Notes

Sequence alignment of *A. castelanii* thioredoxin reductase sequence (Uniprot L8HG59) and *P. falciparum* thioredoxin reductase crystal FASTA sequence (PDB 4J56); alignment was performed with the program BLASTp and the identity between the two sequences was 47.51%; the plus “+” signal represents residue similarity (PDF)
