# Supplementary material for: Amebicidal Effect of Adamantane–Azole Gold(I) Complexes: Cell Death Mechanisms and Synergistic Action with Chlorhexidine against Acanthamoeba castellanii
Source: ACS Omega. 2026 Jan 28;11(5):8361–75. doi: 10.1021/acsomega.5c11190 (PMC12903036; doi:10.1021/acsomega.5c11190)
Supplement: Supplementary file 1 [file ao5c11190_si_001.pdf]

Amebicidal effect of adamantane–azole gold(I) complexes: cell death mechanisms and synergistic action with chlorhexidine against *Acanthamoeba castellanii*

Gabrieli Eduarda Israel[a§], Gabriella da Rosa Monte Machado[a§], Dayara Corrêa Matiola[a], Heveline Silva[b], Lisandra de Oliveira Silva[c], Maico Roberto Luckmann Rodrigues da Silva[d], Suellen dos Reis[e], Vitória Manoela Dambrós[f], Lílian Sibelle Campos Bernardes[e], Evelise Maria Nazari[d], Maria Cláudia Santos-Silva[c], Mário Lettieri Teixeira[f], and Karin Silva Caumo[a]*

[a] Laboratório de Investigação Aplicada a Protozoários Emergentes (LADIPE), Programa de Pós-Graduação em Farmácia, Centro de Ciências da Saúde, Universidade Federal de Santa Catarina, Florianópolis, SC 88040-900, Brazil
[b] Laboratório de Síntese e Interações Bioinorgânicas (SibLab), Departamento de Química, Instituto de Ciências Exatas, Universidade Federal de Minas Gerais, Belo Horizonte, MG 31270-901, Brazil
[c] Laboratório de Oncologia Experimental e Hemopatias (LOEH), Programa de Pós-Graduação em Farmácia, Centro de Ciências da Saúde, Universidade Federal de Santa Catarina, Florianópolis, SC 88040-900, Brazil
[d] Departamento de Biologia Celular, Embriologia e Genética, Centro de Ciências Biológicas, Universidade Federal de Santa Catarina, Florianópolis, SC 88040-900, Brazil
[e] Laboratório de Química Farmacêutica Medicinal, Programa de Pós-Graduação em Farmácia, Centro de Ciências da Saúde, Universidade Federal de Santa Catarina, Florianópolis, SC 88040-900, Brazil
[f] Laboratório de Farmacologia, Instituto Federal Catarinense, Rodovia SC-283, Fragosos, Concórdia, SC 89700-000, Brazil

*Email: k.caumo@ufsc.br

Figure S1

```
A.castelanii  38   EDHTYDYDLIVIGGGSGGLAAAKEAGRLGKKVALLDFVVPTPTGTTWGLGGTCVNVGCIP  97
                   E+HTYDYD +VIGGG GG+A+AKEA   G +V L D+V P+  GT WG+GGTCVNVGC+P
P. falciparum 36   EEHTYDYDYVVIGGGPGGMASAKEAAAHGARVLLFDYVKPSSQGTKWGIGGTCVNVGCVP  95

A.castelanii  98   KKLMHQAALLGESLK-DAQHYGWAVPDNVNHDWEKMVNAVQDHIGSLNWGYRVALREKNV  156
                   KKLMH A  +G   K D++ YGW   DN+ HDW+K+V  VQ HI SLN+ Y   LR   V
P. falciparum 96   KKLMHYAGHMGSIFKLDSKAYGWKF-DNLKHDWKKLVTTVQSHIRSLNFSYMTGLRSSKV  154

A.castelanii  157  NYLNAYGVFVDSHTLECT---DRAKKVTRVTARRFLVATGGRPKYPD-IPGDREFGITSD  212
                    Y+N      D +T+      D +K+ T VT +  L+ATG RP  PD + G +E  ITSD
P. falciparum 155  KYINGLAKLKDKNTVSYYLKGDLSKEET-VTGKYILIATGCRPHIPDDVEGAKELSITSD  213

A.castelanii  213  DFFSLPTPPGKTLVVGASYVALECAGFVRGLGYDTTVMVRSILLRGFDQQLANMIGQYME  272
                   D FSL   PGKTLVVGASYVALEC+GF+  LGYD TV VRSI+LRGFDQQ A  +  YME
P. falciparum 214  DIFSLKKDPGKTLVVGASYVALECSGFLNSLGYDVTVAVRSIVLRGFDQQCAVKVKLYME  273

A.castelanii  273  CHGIKFVRSAVPTKVEKLESGKLRVTFQQDGVEGVEEYDTVMWAIGREAETKKIGLDKAG  332
                     G+ F    +P K+ K++  K+ V F     +  E YDTV++AIGR+ +   + L+
P. falciparum 274  EQGVMFKNGILPKKLTKMDD-KILVEFSD---KTSELYDTVLYAIGRKGDIDGLNLESLN  329

A.castelanii  333  VQVDRIGK--IHTVMERTNVPHIYAIGDIIVDEPSQRSLELTPVAIKAGILLVRRLYAGS  390
                   + V++     I   +  TN+P I+A+GD+  + P     EL PVAIKAG +L RRL+  S
P. falciparum 330  MNVNKSNNKIIADHLSCTNIPSIFAVGDVAENVP-----ELAPVAIKAGEILARRLFKDS  384

A.castelanii  391  TQPMDYINVPTTVFTPIEYGAIGYSEEDAIAQFGEDNLEIYHSYFKPLEWTIAERDDNV-  449
                    + MDY  +PT+++TPIEYGA GYSEE A   +G+ N+E++   F  LE +   R  ++
P. falciparum 385  DEIMDYSYIPTSIYTPIEYGACGYSEEKAYELYGKSNVEVFLQEFNNLEISAVHRQKHIR  444

A.castelanii  450  -------------CYAKLICDKRDSERVVGFHVLGPNAGEITQGFGTAMKAGATKSTFDA  496
                                C AKL+C K +  RV+GFH +GPNAGE+TQG   A++    K  FD
P. falciparum 445  AQKDEYDLDVSSTCLAKLVCLKNEDNRVIGFHYVGPNAGEVTQGMALALRLKVKKKDFDN  504

A.castelanii  497  TVGIHPTTAEEFTTLEVTKRSGV  519
                    +GIHPT AE F  L VT  SG+
P. falciparum 505  CIGIHPTDAESFMNLFVTISSGL  527
```
